# A Large *Plasmodium vivax* Reservoir and Little Population Structure in the South Pacific

**DOI:** 10.1371/journal.pone.0066041

**Published:** 2013-06-18

**Authors:** Cristian Koepfli, Lincoln Timinao, Tiago Antao, Alyssa E. Barry, Peter Siba, Ivo Mueller, Ingrid Felger

**Affiliations:** 1 Swiss Tropical and Public Health Institute, Basel, Switzerland; 2 University of Basel, Basel, Switzerland; 3 PNG Institute of Medical Research, Goroka, Papua New Guinea; 4 Department of Biological Anthropology, University of Cambridge, Cambridge, United Kingdom; 5 Infection & Immunity Division, Walter & Eliza Hall Institute, Parkville, Victoria, Australia; 6 Department of Medical Biology, University of Melbourne, Parkville, Victoria, Australia; 7 Barcelona Centre for International Health Research, Barcelona, Spain; University of Oxford, Viet Nam

## Abstract

**Introduction:**

The importance of *Plasmodium vivax* in malaria elimination is increasingly being recognized, yet little is known about its population size and population genetic structure in the South Pacific, an area that is the focus of intensified malaria control.

**Methods:**

We have genotyped 13 microsatellite markers in 295 *P. vivax* isolates from four geographically distinct sites in Papua New Guinea (PNG) and one site from Solomon Islands, representing different transmission intensities.

**Results:**

Diversity was very high with expected heterozygosity values ranging from 0.62 to 0.98 for the different markers. Effective population size was high (12′872 to 19′533 per site). In PNG population structuring was limited with moderate levels of genetic differentiation. *F*_ST_ values (adjusted for high diversity of markers) were 0.14–0.15. Slightly higher levels were observed between PNG populations and Solomon Islands (*F*_ST_ = 0.16).

**Conclusions:**

Low levels of population structure despite geographical barriers to transmission are in sharp contrast to results from regions of low *P. vivax* endemicity. Prior to intensification of malaria control programs in the study area, parasite diversity and effective population size remained high.

## Introduction

*Plasmodium vivax* is the predominant malaria parasite in many of the countries undergoing concerted efforts to eliminate the disease, and presents a major challenge towards control and elimination of malaria [1]. *P. vivax* is particularly prevalent throughout the South Pacific, including Solomon Islands and Papua New Guinea (PNG), and there are reports of severe outcome of disease, especially from PNG and West Papua [2], [3], [4]. Solomon Islands are among the 32 countries that are eliminating malaria, and the number of confirmed malaria cases decreased by roughly 50% from 2000 to 2010 [5]. PNG is controlling malaria and was the only country in the Western Pacific region with an increase in cases over the last decade [1], [5].

Population structure can inform interventions against malaria. Marked differences in allele frequencies between parasite populations indicate little gene flow, suggesting restricted parasite migration. Likewise, genetic diversity, gene flow and linkage disequlibrium (LD) between loci are predicted to influence the emergence and spread of drug resistance and may affect efficiency of potential future vaccines [6], [7], [8]. Where transmission is already reduced to a low level, genotyping could help to track outbreaks and to identify the origin of imported malaria cases.

Several *P. vivax* population genetic studies from local (village) to intercontinental level were undertaken with samples from Latin America and South-East Asian countries where *P. vivax* prevalence is generally lower than in PNG. Genotyping of microsatellite markers have revealed considerable genetic differentiation between populations, suggesting limited gene flow [9], [10], [11], [12].

*P. vivax* transmission intensity is much higher in PNG compared to that in the Americas and Asia. To investigate *P. vivax* population structure from a setting of high endemicity and to describe its local variations, we have genotyped 14 molecular markers in 295 samples collected in PNG and Solomon Islands prior to the intensification of control activities. Three of the study sites were located in the tropical lowlands of PNG, where transmission is intense. One site located on the southern highland fringe in PNG and a site in Solomon Islands, separated by sea from PNG, were included to study the effect of potential geographical barriers to transmission.

## Methods

### Ethics Statement

This study was performed on an existing sample collection from previous studies. All samples were anonymized prior to use. Approval for this study was obtained from the Institutional Review Board of PNG Institute of Medical Research (amendment to IRB 0919/MRAC 09.24 from the PNG Medical Research Advisory Committee), the Ethics Committee of Canton Basel (no. 237/11) and the National Health Research & Ethics Committee of Solomon Islands (approval no. HRC 12/13). Prior to sample collection, informed written consent was given by all individuals, or in case of children by their parents or guardians.

### Study Sites and Samples

Archived *P. vivax* positive DNA samples collected in the course of earlier studies conducted between 2003 and 2007 were used [13], [14], [15], [16]. At the start of our comprehensive microsatellite typing project, information on multiplicity of infection (MOI) was available for samples from PNG based on the two *P. vivax* genotyping markers MS16 and *msp1*F3 [17]. MS16 showed the highest resolution of all markers, in addition our previous work showed that underestimation of MOI is unlikely when these two markers are typed [18]. Results from MS16 and *msp1*F3 thus provided the basis for the selection of preferentially single clone infections or low multiplicity samples for further analysis. Details of samples used in this study and their origin are listed in Table 1.

**Table 1:** *P. vivax* samples included in this study.

Site	year of collection	Age of study participants	no. of *P. vivax* pos. samples collected	Mean MOI of all*P. vivax* pos. samples^a^	Proportion of multiple clone infections in all *P. vivax* pos. samples^a^	no. of samples genotyped^b^	References
Ilaita	2006–2007	0.9–4.5 years	2096	2.67	73.1%	132	[15], [17]
Kunjingini	2003–2005	0.5–7 years	94	2.07	63.8%	38	[13]
Alexishafen	2005–2007	0.5–5 years	150	2.27	72.7%	45	[14], [22]
Sigimaru	2004–2005	0.5–7 years	48	2.6	70.8%	39	[13]
Solomon Islands	2004–2005	>6 months^c^	68	2.75	88.3%	41	[16]

a based on our initial typing results obtained with 2 loci, *msp1*F3 and MS16.

b number of samples included in the final data set after successful amplification of 12 microsatellite markers (in addition to MS16 and *msp1*F3).

c includes adults and children above 6 months of age.

**Figure 1:**
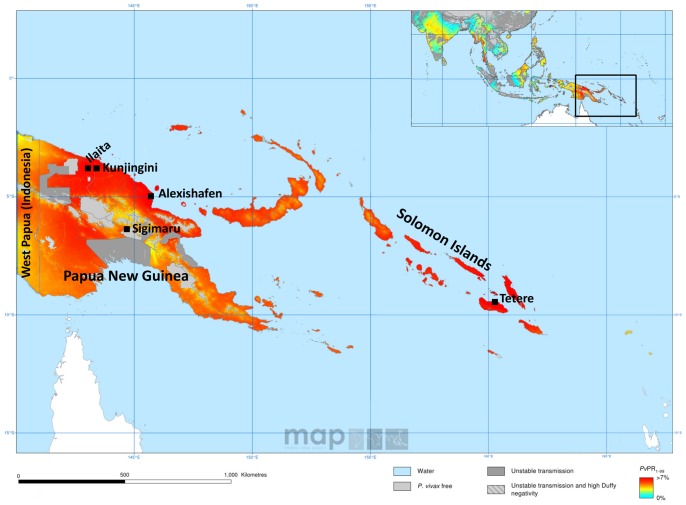
Spatial distribution of *P. vivax* endemicity in Papua New Guinea and Solomon Islands in 2010 and origin of *P. vivax* positive blood samples analyzed in this study. Colors indicate the model-based geostatistic point estimates of the *P. vivax* annual parasite incidence (*PvAP*I) in 2010 in the 1–99 years age range [53]. Unstable transmission (medium grey areas) is defined as *PvAPI* <0.1 per 1000 individuals per year. Squares indicate the origin of genotyped *P. vivax* samples from PNG and Solomon Islands.

295 *P. vivax* positive blood samples from four sites in PNG and one site on Solomon Islands were included in the study. Three of the study sites in PNG were located in the hyper- to holoendemic tropical lowlands: Ilaita (n = 132 samples) and Kunjingini (n = 38, both Maprik District, East Sepik Province) and Alexishafen (n = 45, Madang Province). The fourth PNG study site was in a meso-endemic site at the Southern highlands fringe at an altitude of 1100 metres (n = 39, Sigimaru, Karimui area, Simbu Province) (Figure 1).

Large parts of the coastal lowlands of PNG are characterized by high prevalence of *P. vivax* and *P. falciparum* with perennial transmission and mild seasonal variation. In study participants from the Ilaita site, prevalence by microscopy was 44.3% for *P. vivax*, 32.6% for *P. falciparum* and 4.2% of *P. malariae*. The incidence rate of malaria was 2.46 episodes per child per year for *P. vivax* and 2.56 for *P. falciparum* [15]. Malaria prevalence is lower in the highland fringe of Simbu province. In a survey in 2001–2002 prevalence was 8% for *P. vivax* and 27% for *P. falciparum* in South Simbu, where our study site is located [19].

Solomon Islands samples (n = 41) derived from asymptomatic children >6 months and adults from the Tetere area (Guadalcanal Province), collected in 2004 and 2005 [16]. At that time transmission of *P. falciparum* and *P. vivax* in the area was considered mesoendemic. *P. vivax* prevalence by microscopy was 19.1% compared to a *P. falciparum* prevalence of 12.9% ([16] and Marie Ballif, unpublished results).

Preliminary analysis of longitudinal data from the Ilaita cohort showed no changes in diversity or structuring of samples over time. Thus, the collection of samples in different years is not expected to influence the results.

### Genetic Markers and PCR

To allow comparability with previous studies from different continents [10], [12] a panel of 13 well-described and frequently used size polymorphic *P. vivax* markers were selected for genotyping: MS1, MS2, MS5, MS6, MS7, MS8, MS9, MS10, MS12, MS15, MS16, MS20 [20], Pv3.27 [9], [21]. In addition the polymorphic F3 region of the *merozoite surface protein 1* (*msp1*F3) was typed [17], [21]. *msp1*F3 codes for a size polymorphic domain of the surface antigen MSP1 and could be under balancing selection. To assess the effect of a potentially non-neutral marker, we have calculated *F*_ST_ values including and excluding *msp1*F3, and excluded *msp1*F3 from all further analyses.

For MS16 and *msp1*F3, genotyping results were available from earlier studies [17], [21], [22], except for samples from Solomon Islands, which were typed as described [17]. For the 12 additional microsatellite markers a semi-nested PCR protocol with 12-plex primary PCR followed by individual reactions for nested PCR was applied. Forward primers for the 12 primary PCR assays were designed and used in combination with previously published nested reverse primers [9], [20] (file S1). PCR products were sized by capillary electrophoresis and analysed as described [17].

Linkage disequilibrium estimation and analysis of population structure by STRUCTURE software requires genotyping data from multi-locus haplotypes. Up to 88% of all individuals in our study sites carried multiple clone infections (Table 1). From these infections we included only the predominant clone (based on peak heights) of each sample into our analysis, all minority clones were ignored. During method optimization we have genotyped field samples in triplicate [17]. These experiments showed that the predominant clone is maintained through nested PCR with only minor variation in peak height ratios. The same was observed during amplification of artificial mixtures of parasite strains [23]. To account for this minor variation we have excluded all results from those markers where the minor allele exceeded 70% of the peak height of the dominant peak.

### Data Analysis

Alleles were binned into 2, 3 or 4 bp bins according to their repeat unit size using TANDEM 1.08 software [24]. The software Lositan was used to detect loci that are under positive or balancing selection. To detect non-neutral loci 100’000 simulations were run in Lositan under a stepwise mutation model [25], [26]. Lositan software gave no indication of non-neutral loci (including *msp1*F3), and all markers were included into further analysis.

The expected heterozygosity *H*_E_ was determined as a measure for genetic diversity and was calculated for each marker as follows:
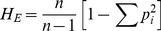
where n is the number of clones and p_i_ the frequency of allele i. Linkage disequilibrium (LD) was assessed using the program LIAN 3.5 applying a Monte Carlo test with 100’000 random resamplings [27]. LIAN cannot handle missing data, thus 6 markers and subsequently all samples still containing missing data were removed from the data set. The remaining dataset included 165 samples and the markers MS2, MS7, MS9, MS10, MS12, MS15, MS16 and MS20 (Table 2). In addition, a reduced dataset (107 samples) including only samples with 1 allele detected for these 8 markers was analysed. To confirm that LD estimates were not influenced by selection of markers, we have re-run different combinations of markers and found similar results. The standardized index of association *I_A_^S^* was calculated as an estimation of LD. *I_A_^S^* is zero for linkage equilibrium.

**Table 2:** Linkage disequilibrium between 8 markers (MS2, MS7, MS9, MS10, MS12, MS15, MS16, MS20) determined by LIAN software.

	All samples			MOI = 1		
	# samples	*I_A_^S^*	*P*	# samples	*I_A_^S^*	*P*
Ilaita	74	0.0049	0.131	47	0.0065	0.143
Kunjingini	23	0.021	0.0557	17	0.0164	0.183
Alexishafen	35	−0.0001	0.516	30	−0.0001	0.515
Sigimaru	22	0.0248	0.0422	12	0.1073	0.00028^a^
Solomons	11	−0.0135	0.758	NA	NA	NA
All	165	0.0032	0.0739	107	0.0036	0.113

a Linkage in Sigimaru was not significant if only unique haplotypes were analyzed (11 samples, *I_A_^S^* = 0.0037, *P* = 0.400.

To compare allelic frequencies between populations, Weir & Cockerham *F_ST_* values were calculated using Fstat version 2.9.3.2 [28]. In the case of highly diverse markers such as microsatellites, *F_ST_* values are downwardly biased [29]. To ensure comparability to other studies we have calculated in addition corrected *F*_ST_ values that are adjusted for the high diversity of our markers using RecodeData 0.1 [30]. As a complementary approach to assess population structure, we have analysed haplotypes using STRUCTURE 2.3.2 [31]. This algorithm attempts to form groups of haplotypes without prior information on the origin of a sample and is able to detect genetic differences among subgroups that do not correlate with geography. The number of populations (K) was set from 1 to 10 with 3 replications per K, each with 100’000 Markov Chain Monte Carlo steps after a burn-in period of 10′000 steps, using the admixture model. In order to determine the most probable number of populations, ΔK was calculated, i.e. the change in the posterior probability of the data for a given K [32].

Principal component analysis (PCA) was done in R using the prcomp function. Haplotypes containing missing data were excluded to calculate the covariance matrix, but projected into the final plot if not more than three data points were missing.

We estimated the long-term effective population size (N_e_) using a similar procedure as for *P. falciparum* [33]. For three markers (MS8, MS16, Pv327) the number of different alleles detected was similar to the number of samples taken. Furthermore, in some populations the number of samples was even lower than the total number of different alleles sampled across all populations. A low number of samples compared to the number of alleles present across all populations biases the N_e_ estimates, resulting in values that are incorrectly high. We thus excluded MS8, MS16 and Pv3.27 and used only the remaining 10 markers to estimate effective population size. As estimates of microsatellite mutation rates (μ) are not available for *P. vivax*, we used estimates for *P. falciparum* of 1.59×10^−4^ (95% confidence interval: 6.98×10^−5^, 3.7×10^−4^) [34]. For a pure step-wise mutation model (SMM), N_e_ is given by
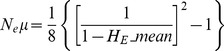
where *H*_E__mean is the mean expected heterozygosity across all loci. As some loci showed deviation from the SMM model, we also estimated N_e_ using the formula for the infinite allele model (IAM):



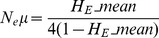



## Results

We have genotyped 14 size polymorphic molecular markers in 295 *P. vivax* positive blood samples from different locations in PNG and Solomon Islands. All markers were highly polymorphic with 8–81 alleles per locus and *H*_E_ values of 0.69–0.98. Levels of diversity were similar at all sites (Table 3). Within all populations, 294 different haplotypes were detected, with one haplotype detected twice in Sigimaru. No linkage disequilibrium (LD) was observed in the data, except for samples from Sigimaru in the PNG highlands (Table 2). No LD was observed in samples from Sigimaru when only unique haplotypes were analyzed.

**Table 3:** Characteristics and expected heterozygosity *H*_E_ of 14 *P. vivax* genotyping markers overall and separately for 5 populations from PNG and Solomon Islands.

Marker	Chromo-some	size range (bp)	no. of alleles	expected heterozygosity *H*_E_					
				Overall	Ilaita	Kunjingini	Alexishafen	Sigimaru	Solomons
MS1	3	221–251	9	0.69	0.67	0.69	0.70	0.75	0.71
MS2	6	168–380	24	0.91	0.91	0.93	0.93	0.89	0.89
MS5	6	143–197	15	0.88	0.86	0.85	0.89	0.84	0.77
MS6	11	210–255	12	0.85	0.85	0.88	0.85	0.74	0.83
MS7	12	138–243	17	0.79	0.75	0.80	0.83	0.78	0.87
MS8	12	181–334	39	0.96	0.97	0.96	0.96	0.95	0.93
MS9	8	152–173	8	0.80	0.77	0.86	0.87	0.77	0.74
MS10	13	156–213	20	0.90	0.90	0.88	0.86	0.90	0.74
MS12	5	168–231	10	0.69	0.65	0.62	0.71	0.70	0.80
MS15	5	231–291	20	0.89	0.87	0.87	0.90	0.89	0.84
MS16	9	194–572	81	0.98	0.98	0.97	0.97	0.97	0.90
MS20	10	158–251	28	0.93	0.91	0.91	0.90	0.92	0.92
Pv3.27	3	184–460	33	0.93	0.93	0.93	0.94	0.93	0.84
*msp1*F3	7	237–372	30	0.84	0.83	0.74	0.90	0.88	0.72
Mean			24.7	0.861	0.845	0.849	0.872	0.851	0.822

We have compared the 5 populations studied by calculating *F*_ST_ values. Within PNG, uncorrected values were very low (*F*_ST_ = 0 to 0.016, Table 4A), and remained moderate after correcting for high diversity of markers (*F*_ST_ = 0.14). The population genetic difference between lowland sites and Sigimaru was not higher than among lowland sites. Uncorrected *F*_ST_ values ranged from 0.030 to 0.044 between Solomon Islands and PNG, corrected values ranged from 0.15 to 0.16 (Table 4A). Differences between all populations were statistically significant when using the corrected data set (*P*<0.005).

**Table 4:** Genetic differentiation of *P. vivax* populations in Papua New Guinea and Solomon Islands.

A) 13 microsatellites					
	Ilaita	Kunjingini	Alexishafen	Sigimaru	Solomons
Ilaita		−0.002	**0.010** *	**0.013** *	**0.041** *
Kunjingini	**0.148** *		0.002	0.008	**0.049** *
Alexishafen	**0.142** *	**0.136** *		**0.019** *	**0.033** *
Sigimaru	**0.152** *	**0.147** *	**0.140** *		**0.050** *
Solomons	**0.160** *	**0.156** *	**0.149** *	**0.160** *	
**B) Marker ** ***msp1*** **F3 alone**					
	**Ilaita**	**Kunjingini**	**Alexishafen**	**Sigimaru**	**Solomons**
Ilaita		−0.006	**0.035** *	**0.011** *	**0.046** *
Kunjingini	**0.209** *		0.055	0.013	0.023
Alexishafen	**0.138** *	**0.173** *		0.007	**0.064** *
Sigimaru	**0.151** *	**0.189** *	**0.110** *		0.022
Solomons	**0.217** *	**0.268** *	**0.181** *	**0.198** *	
**C) Combination of 13 microsatellites and ** ***msp1*** **F3**					
	**Ilaita**	**Kunjingini**	**Alexishafen**	**Sigimaru**	**Solomons**
Ilaita		−0.002	**0.012** *	**0.013** *	**0.042** *
Kunjingini	**0.153** *		0.006**	0.008**	**0.048** *
Alexishafen	**0.142** *	**0.139** *		**0.018** *	**0.035** *
Sigimaru	**0.152** *	**0.150** *	**0.138**		**0.048** *
Solomons	**0.164** *	**0.164** *	**0.151**	**0.163** *	

*F*_ST_ values for inter-site comparisons are given. The upper-right section shows uncorrected values. In the lower-left section *F*_ST_ values are corrected to adjust for high diversity of our microsatellite markers (see methods for details).

(A) results obtained from 13 microsatellite markers.

(B) results obtained from marker *msp1*F3 only.

(C) results obtained from 13 microsatellite markers and *msp1*F3.

* significant at the Bonferroni-corrected 5% level (*P*<0.005).

** significant at the 5% level (*P*<0.05).

**Figure 2:**
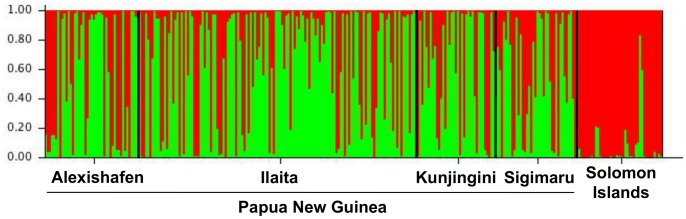
Output of clustering analysis by STRUCTURE Software for 2 clusters (K = 2). Each column represents one haplotype, the green and red colours show whether an isolate was assigned to cluster 1 or cluster 2. If both colours are present, the haplotype consists of a mixture of markers assigned to cluster 1 and to cluster 2. The samples from Solomon Islands were assigned mostly to one cluster (red), while samples from PNG contain alleles of both clusters.

**Figure 3:**
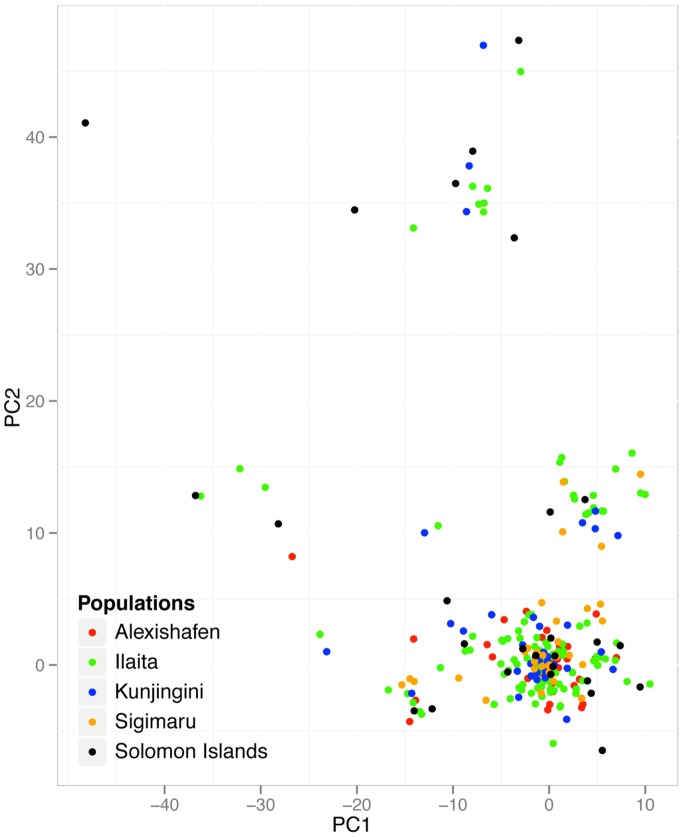
Principal component analysis of *P. vivax* haplotypes genotyped with 13 microsatellite markers in 4 populations from PNG and one site in Solomon Islands. Haplotypes were clustered to show maximal differentiation. The first principal component (PC1) has the largest possible variance (i.e. it accounts for as much of the variability in the data as possible), the second principal component (PC2) has the second largest possible variance. Isolates do not cluster according to their geographic origin, indicating absence of pronounced population structure.

As an alternative approach to assess population structure we have searched for clustering of genotypes without prior information on the origin of samples using STRUCTURE software. The most likely number of clusters (K) was 2, with separation between samples from PNG and Solomon Islands and thus following geographical structure (Figure 2). No sub-structuring was found within PNG as STRUCTURE is not adequate for detection of very low, yet significant *F*_ST_ values [35]. Principal component analysis did not reveal any grouping of haplotypes according to their origin, further confirming absence of marked population structure (Figure 3).

We have calculated effective population size in the sampled populations. N_e_ (with SMM) ranged from 12,872 to 22,528 (Table 5). When all populations from PNG were pooled (a reasonable assumption as populations were genetically similar), N_e_ was 25,919. Due to the very high diversity of the populations studied confidence intervals were large. Estimates based on IAM were around 3 times lower.

**Table 5:** Effective population size N_e_ per study site and for PNG populations pooled. Estimates are based on 10 microsatellite markers (MS1, MS2, MS5, MS6, MS7, MS9, MS10, MS12, MS15, MS20).

	PNG	Ilaita	Kunjingini	Alexishafen	Sigimaru	Solomons
SMM [95% CI]	25919[11138–59043]	19533[8394–44496]	15970 [6862–36379]	22528 [9681–51317]	14716 [6324–33523]	12872 [5531–29322]
IAM [95% CI]	7591 [3262–17293]	6421 [2759–14627]	5686 [2443–12953]	6990 [3003–15923]	5409 [2324–12323]	4981 [2140–11347]

Estimates are calculated using a stepwise mutation model (SMM) and an infinite allele model (IAM), and are given for each population from PNG and Solomon Islands separately as well as for samples from PNG pooled.

In addition to the 13 presumably neutral microsatellite markers, we have genotyped the marker *msp1*F3, which encodes intragenic repeats of the *P. vivax* merozoite surface protein MSP1. This marker might be subject to selection. We have calculated *F*_ST_-values for *msp1*F3 alone (Table 4B) and for a combined dataset of the 13 microsatellites and *msp1*F3 (Table 4C). *F*_ST_ values were similar for all data sets. No indication for balancing selection for *msp1*F3 was found. Instead differences in *msp1*F3 allelic frequencies between PNG and Solomon Islands were observed. The predominant allele in PNG of 265 bp in size reached a frequency of 0.24 in the Ilaita cohort [17], whereas in Solomon Islands its frequency was 0.48. In contrast, the second most frequent allele in the Ilaita cohort (238 bp in size, frequency = 0.17) was detected in Solomon Islands a single time, only when also minority clones were included and thus the number of clones was increased to 99.

## Discussion

Four human *Plasmodium* species, *P. falciparum*, *P. vivax*, *P. malariae* and *P. ovale*, coexist in PNG, with probably the highest *P. vivax* prevalence anywhere in the world. While *P. vivax* prevalence was 44% in the Ilaita study site, it was 19% in neighboring Solomon Islands.

The population genetic structure from these South Pacific sites is in sharp contrast to the one in Latin America, where *P. vivax* is the predominant malaria parasite, but endemicity is usually low [9], [10], [11], [12]. Within PNG and Solomon Islands the genetic composition of *P. vivax* populations differed only moderately with *F*_ST_ values around 0.15, despite geographical barriers to transmission, i.e. largely malaria free mountain ranges between Sigimaru and the lowland sites in PNG and a distance of up to seventeen hundred km between sampling sites in PNG and Solomon Islands. In contrast *F*_ST_ values were 0.4–0.7 between five sites in Colombia [9]. Even on a scale of 2 to 50 km distance between sites in Peru high levels of population differentiation were found [10]. *P. vivax* is also the predominant *Plasmodium* species in many Asian countries, inter-country *F*_ST_-values ranged from 0.13–0.45 between populations from India, Laos and Thailand [9]. Differentiation between *P. vivax* populations in PNG was low also in comparison to sympatric *P. falciparum* populations from lowland PNG (uncorrected *F*_ST_-values *P. falciparum*: 0.05–0.14 [36] vs. *P. vivax* 0.030 to 0.044). Microsatellite diversity in Solomon Islands was similar to PNG, despite its relative geographical isolation. The moderate *P. vivax* population structure in the South-West Pacific is contrasting the pattern observed in the human population that revealed major differences between cultural and linguistic groups on small geographical scales [37].

Differences in transmission levels, effective population size, geographical isolation, recent control and elimination efforts and the history of malaria radiation may all contribute to the difference in *P. vivax* population structure between the South-West Pacific, South America and South-East Asia. The origin of *P. vivax* in South America is unclear. Malaria was likely brought to the Amazon by European conquerors 1492 onwards and continuing slave trade [38]; however an earlier independent introduction from Melanesia to the Pacific coast cannot be ruled out. The current substructure might be a consequence of independent introductions, especially since *P. vivax* in the Americas has never reached a prevalence as high as in PNG, and it was close to elimination a few decades ago. In the South Pacific *P. vivax* arrived much earlier than in the Americas, possibly together with the first human settlers 30′000 to 50′000 years ago and was the predominant malaria species prior to the start of malaria control in the second half of the 20^th^ century [39]. The samples used in this study were collected prior to recently intensified malaria control interventions and thus reflect a situation of intense transmission. While in PNG combination therapy with chloroquine or amodiaquine plus sulfadoxine–pyrimethamine was introduced in 2000 [39], large scale distribution of insecticide treated bed nets has only started after sample collection for this study. In Solomon Islands, malaria control largely ceased during the civil unrest between 1998 and 2002 and was only intensified in 2003 [5].

We estimated very high values for the effective population size, comparable or above the *P. falciparum* N_e_ in areas of high endemicity [33], [40], [41], confirming recent suggestions that *P. vivax* exhibits a larger effective population size than *P. falciparum* [42]. Estimates of effective population size based on heterozygosity reflect long term processes because *H*_E_ is changing very slowly over time. Therefore *H*_E_ is not a reliable indicator for studying contemporary demographic processes. We also tried to estimate contemporary N_e_ using LD estimation [43], [44], but all estimates provided an infinite population size. It is known from theoretical expectations [43] and empirical studies [45] that high N_e_ reduces the precision of estimates and infinite values are likely. Despite the difficulty to compute contemporary N_e_, it is clear that N_e_ is very high in all our population.

High N_e_ reduces the rate of genetic drift and thus the amount of gene-flow required to counteract population differentiation. Further aspects of *P. vivax* biology facilitate break down of population structure despite a relatively low level of human migration within PNG and between PNG and Solomon Islands. Firstly, *P. vivax* can relapse from dormant liver-stages months or years after the initial infection. Secondly, clinical immunity to *P. vivax* is acquired very rapidly. Under high exposure the incidence of clinical *P. vivax* peaks in children below 2 years and by 5 years of age children in PNG have acquired almost complete clinical immunity to *P. vivax* [15], [46]. The prevalence of asymptomatic infections however remains high even in adults [47]. Similarly high rates of asymptomatic infections are also observed in Solomon Islands [48]. *P. falciparum* is less latent and thus shows a higher degree of population structure. Differences between the two species are also evident in the highlands of PNG, where high diversity of *P. vivax* reflects endemic transmission, while clonal *P. falciparum* population structure suggests on outside introduction [49]. Because of the high diversity of our microsatellite markers, a more fine-scale *P. vivax* population structure within PNG could have escaped detection, but might be revealed using slower evolving markers such as SNPs.

As a combined result of intense transmission, relapses and acquired immunity many individuals carry multiple clone *P. vivax* infections [17], which might affect *P. vivax* population structure. Sexual recombination is only possible in the mosquito after uptake of gametocytes of different parasite strains. This is more likely in the case of high multiplicity in the human host. Low levels of sexual recombination result in clonal population structure with high LD and possibly a high degree of separation between nearby populations. A high frequency of recombination breaks LD and is expected when transmission is intense and parasite genotypes circulate between different populations. In our study populations in PNG and Solomon Islands, the proportion of people carrying multiple clone infections ranged from 63% to 88% (Table 1). In areas of low *P. vivax* transmission this proportion ranged from 13% in Sri Lanka [50] to 10–34% in South America [9], [10] or 30–35% in Thailand and Laos [9]. Absence of LD in our data (with the exception of Sigimaru, where inclusion of a haplotype occurring twice lead to significant LD) further implies a high frequency of sexual recombination. High LD was reported previously from low transmission areas, such as the Amazon [9], [10], [51], Sri Lanka or Ethiopia [11], [12].

The high proportion of *P. vivax* multi-clone infection contrasts with the situation in *P. falciparum* populations from lowland PNG with moderate population structuring, where 39–44% of infected individuals carried multiple clones [36]. In the Ilaita cohort, from which part of our samples derived, 33% of *P. falciparum* positive children carried multiple clone infections (Sonja Schoepflin, unpublished), in contrast to 73% multiple-clone infections for *P. vivax* [17].

While our 13 microsatellite markers are presumable neutral, *msp1*F3 encodes a highly polymorphic region of the MSP1 antigen. Our amplified fragment is located approximately 1 kb upstream of the highly antigenic C-terminal MSP1_19_ fragment which is a widely studied vaccine candidate (reviewed in [52]). Consequently, balancing selection could be expected to act on *msp1*F3, but we did not detect evidence for this in our data.

### Conclusions

We present the first comparison of *P. vivax* populations in the South Pacific based on genotyping results from neutral markers. At all study sites we observed very high diversity of parasite genotypes and very high population sizes. Only moderate population sub-structuring was observed even after correcting for the high diversity of markers, despite geographic and social barriers among our study sites. Solomon Islands and Vanuatu represent the south-western boarder of global malaria transmission, and restricted parasite diversity and increasing sub-division between populations is expected towards the spatial limits of malaria endemicity. At the time of sample collection this effect was not evident in Solomon Islands.

Due to the high population size, we cannot precisely estimate the amount of gene flow among our study sites. Further research is warranted to investigate how the continued intensification of malaria control affects the levels of *P. vivax* population differentiation across an extended geographic region, e.g. including Indonesia, Malaysia and the Philippines. Once prevalence is substantially reduced, future attempts to control malaria transmission in regions of previously high *P, vivax* endemicity need to carefully consider migration of infected hosts, even in island settings. Our results illustrate that control programs coordinated between countries and also targeting asymptomatic carriers seem to be needed in order to move towards elimination of *P. vivax* in the South Pacific.

## Supporting Information

File S1 **PCR conditions to amplify 12 microsatellite markers.**
(DOC)
