# Supplementary material for: A Large Plasmodium vivax Reservoir and Little Population Structure in the South Pacific
Source: PLoS One. 2013 Jun 18;8(6):e66041. doi: 10.1371/journal.pone.0066041 (PMC3688846; doi:10.1371/journal.pone.0066041)
Supplement: File S1 — PCR conditions to amplify 12 microsatellite markers. (DOC) [file pone.0066041.s001.doc]

**Supporting File S1: PCR Conditions**

**Primary PCR**

Multiplex primary PCR for 12 microsatellites (MS1, MS2, MS5, MS6, MS7, MS8, MS9, MS10, MS12, MS15, MS20, Pv3.27) was performed in a total volume of 50 μl under the following conditions optimized for multiplex reactions:

Reaction mix

| Template DNA | 1 μl | Supplier |
| --- | --- | --- |
| Buffer B | 5 μl | Solis Biodyne |
| dNTPs | 0.3 mM | Solis Biodyne |
| MgCl_2_ | 3 mM | Solis Biodyne |
| Primer, each of 24 | 0.25 μM | MWG Operon |
| *Taq* Firepol | 10 U | Solis Biodyne |
| dH_2_O | Add to 50 μl |  |

Cycling conditions

| 95°C | 1 min |
| --- | --- |
| 95°C | 15 sec |
| 60°C | 30 sec |
| 72°C | 30 sec ⇒ 24 times back to step 2 |
| 72°C | 5 min |

**Nested PCR**

Primary PCR product was diluted 1:40 in H_2_O and 1μl was used as template in nested PCR. Individual nested PCRs were conducted in a total volume of 20 μl under the following conditions:

Reaction mix

| Diluted primary PCR | 1 μl | Supplier |
| --- | --- | --- |
| Buffer B | 2 μl | Solis Biodyne |
| dNTPs | 0.2 mM | Solis Biodyne |
| MgCl_2_ | 4 mM | Solis Biodyne |
| Primer, each | 0.25 μM | Applied Biosystems |
| *Taq* Firepol | 1.5 U | Solis Biodyne |
| dH_2_O | Add to 20 μl |  |

Cycling conditions

| 95°C | 1 min |
| --- | --- |
| 95°C | 15 sec |
| 61°C | 30 sec |
| 72°C | 45 sec ⇒ 34 times back to step 2 |
| 72°C | 5 min |

Primer sequences

| Marker | Primer name | Sequence 5' - 3' | Fluorescent dye |
| --- | --- | --- | --- |
| MS1 | primary forward | CATCTCGACATGTCGACGTAG |  |
|  | nested forward | TCAACTGTTGGAAGGGCAAT | 6-FAM |
|  | reverse | ctgtcttTTGCTGCGTTTTTGTTTCTG |  |
| MS2 | primary forward | AGCACGACCAACAAGAGAGG |  |
|  | nested forward | GAGCTAGCCAAAGGTTCAACA | VIC |
|  | reverse | ctgtcttTGGGGAGAGACTCCCTTTTC |  |
| MS5 | primary forward | TTCGGCTGGTTTCCAATTAGG |  |
|  | nested forward | CGTCCTCTATCGCGTACACA | NED |
|  | reverse | ctgtcttAAAGGGAGAGGAGCGAAAAC |  |
| MS6 | primary forward | GAGCTGCTGCTTCTATTTTGGG |  |
|  | nested forward | GGTTCTTCGGTGATCTCTGC | VIC |
|  | reverse | ctgtcttGGAGGACATCAACGGGATT |  |
| MS7 | primary forward | ACATCAAAGCAAAGAAGAGGG |  |
|  | nested forward | TTGCAGAAAATGCAGAGAGC | 6-FAM |
|  | reverse | ctgtcttAGGGTCTTCAGCGTGTTGTT |  |
| MS8 | primary forward | AAACGTAAAACCTTTGGCGG |  |
|  | nested forward | AGAGGAGGCAGAAATGCAGA | NED |
|  | reverse | ctgtcttAGCCCCTTTGCGTTCTTTAT |  |
| MS9 | primary forward | TGAATTTCCCCATTTGCCCG |  |
|  | nested forward | AGATGCCTACACGTTGACGA | VIC |
|  | reverse | ctgtcttGAAGCTGCCCATGTGGTAAT |  |
| MS10 | primary forward | AGGACCAAACGGAGGACATG |  |
|  | nested forward | TTATCCCTGCTGGATGTGAA | 6-FAM |
|  | reverse | ctgtcttTCCTTCAGGTGGGACTTGTT |  |
| MS12 | primary forward | AACGTTTCCTTGCCCACTTG |  |
|  | nested forward | AATGCGCATCCTATGTCTCC | NED |
|  | reverse | ctgtcttCTGCTGTTGTTGTTGCTGCT |  |
| MS15 | primary forward | CGCACTCTTCATCCTCATCG |  |
|  | nested forward | TGTTTGCAAAGGAATCCACA | VIC |
|  | reverse | ctgtcttCGGCCAGATGAAAAGGATAA |  |
| MS16 | primary forward | TTCCTGATGACAATTTCGACGG |  |
|  | primary reverse | TCTCTTCCCATTTGAGCATCGC |  |
|  | nested forward | CTTGTTGTGGTTGTTGATGGTG | VIC |
|  | nested reverse | ctgtcttAGTACGTCAACCATGTGGGTAG |  |
| MS20 | primary forward | CAAGGTGCGATGGAAGATTGG |  |
|  | nested forward | GCACAACAAATGCAAGATCC | VIC |
|  | reverse | ctgtcttGTGGCAGTGGCTCATCTTCT |  |
| Pv3.27 | fwd primary | TTTTTCAACTTGCTGCCCCCTG |  |
|  | fwd nested | GGACATTCCAAATGTATGTGCAGTCG | 6-FAM |
|  | reverse | ctgtcttCGTCATCGTCATTGCTCTGGAG |  |
| *msp1*F3 | primary forward | GGAGAACATAAGCTACCTGTCC |  |
|  | primary reverse | GTTGTTACTTGGTCTTCCTCCC |  |
|  | nested forward | CAAGCCTACCAAGAATTGATCCCCAA | VIC |
|  | nested reverse | ctgtcttATTACTTTGTCGTAGTCCTCGGCGTAGTCC | |

In case that only one reverse primer sequence is given, a semi-nested protocol was used with identical reverse primers for both primary and nested PCR. A 7-pb tail promoting addition of a terminal adenine to amplification product is given in lower-case letters.

**Capillary Electrophoresis**

5 μl of each nested amplification product were run on a 1.5% agarose gel. According to the intensity of bands on this gel, PCR products were diluted in dH_2_O either in a ratio of 1:10, 1:20 or 1:40. 2.5 μl of the diluted amplification product was analysed by capillary electrophoresis (CE) together with size standard Rox-500 (Applied Biosystems).
